# First-trimester vaginal microbiome diversity: A potential indicator of preterm delivery risk

**DOI:** 10.1038/s41598-017-16352-y

**Published:** 2017-11-23

**Authors:** Mohammed Monzoorul Haque, Mitali Merchant, Pinna Nishal Kumar, Anirban Dutta, Sharmila S. Mande

**Affiliations:** Bio-Sciences R&D Division, TCS Research, Tata Consultancy Services Ltd., Pune, 411013 India

## Abstract

Preterm birth is a leading cause of global neonate mortality. Hospitalization costs associated with preterm deliveries present a huge economic burden. Existing physical/biochemical markers for predicting preterm birth risk are mostly suited for application at mid/late pregnancy stages, thereby leaving very short time (between diagnosis and delivery) for adopting appropriate intervention strategies. Recent studies indicating correlations between pre/full-term delivery and the composition of vaginal microbiota in pregnant women have opened new diagnostic possibilities. In this study, we performed a thorough meta-analysis of vaginal microbiome datasets to evaluate the utility of popular diversity and inequality measures for predicting, at an early stage, the risk of preterm delivery. Results indicate significant differences (in diversity measures) between ‘first-trimester’ vaginal microbiomes obtained from women with term and preterm outcomes, indicating the potential diagnostic utility of these measures. In this context, we introduce a novel diversity metric that has significantly better diagnostic ability as compared to established diversity measures. The metric enables ‘early’ and highly accurate prediction of preterm delivery outcomes, and can potentially be deployed in clinical settings for preterm birth risk-assessment. Our findings have potentially far reaching implications in the fight against neonatal deaths due to preterm birth.

## Introduction

Technological advances in medical diagnostics and therapeutics in the last decade have greatly reduced the burden of several life-threatening diseases affecting young children^1^. The Millennium Development Goals (MDG) report released by UN in 2015 indicates significant reduction in the incidence rates of malaria, tuberculosis, measles, and AIDS in the last 15 years^2^. However, amidst these encouraging signs of improvement, the report highlights that a majority of deaths in children (under 5) occur within the first 28 days of life (i.e. the neonatal period). Complications arising due to preterm births are indicated as the single largest contributor to neonatal deaths^3,4^. Surprisingly, in comparison to the decrease in incidence rates of other diseases across the globe, the rate of preterm births has remained more or less constant, irrespective of a country’s economic status. For instance, in 2014, the rate of preterm births in US stood at 9.6%, which is quite comparable to the rate (~12%) tracked in countries from the developing world (Brazil, India, and Nigeria)^5^. Overall, these trends highlight the urgent need for greater international attention on developing improved methods for diagnosis, prevention, and management of preterm births.

**Figure 1:**
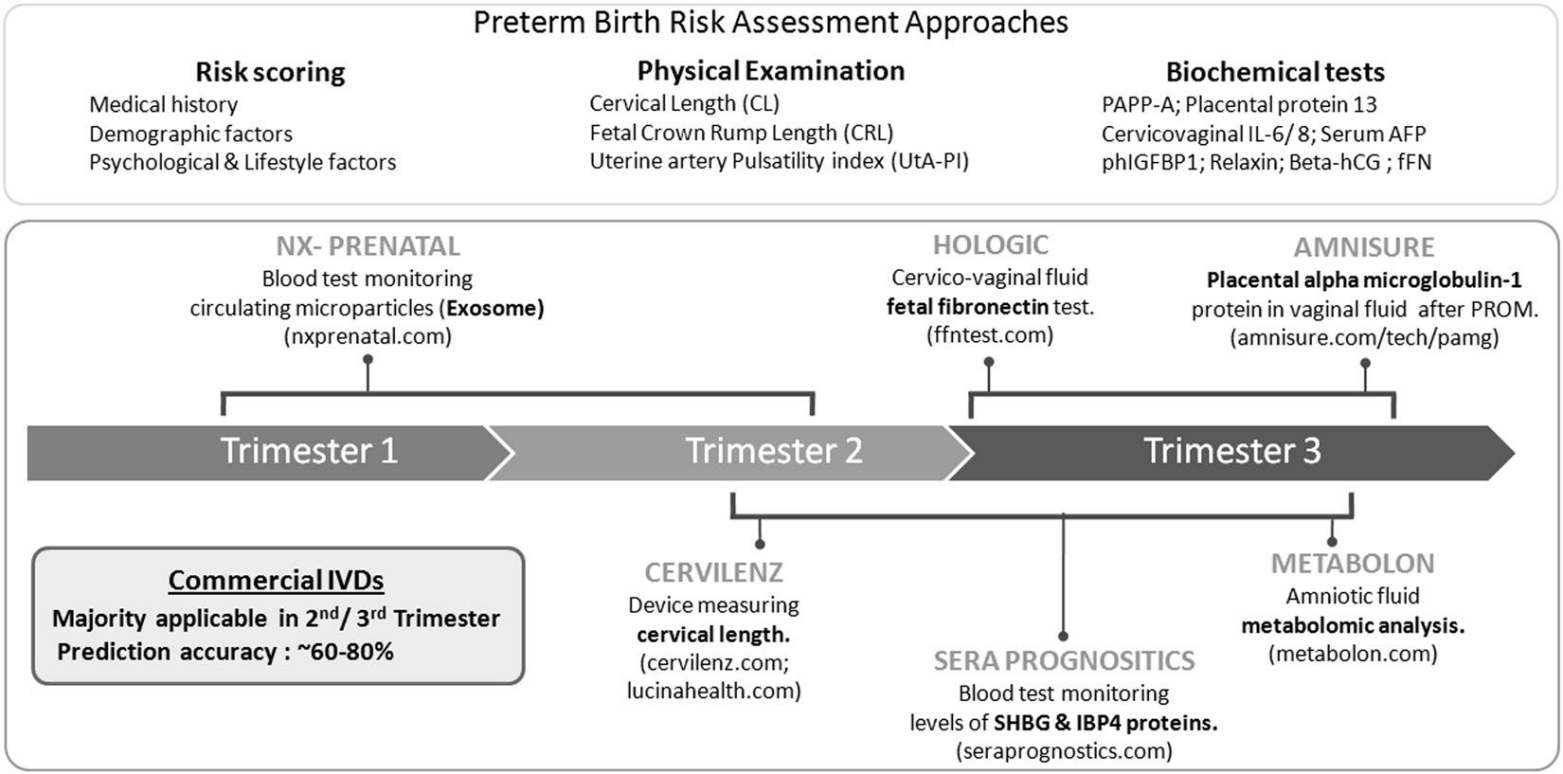
An overview of approaches (and few commercial IVDs i.e. *in vitro* diagnostic offerings) that are used for determining preterm delivery risk.

The entire cascade of patho-physiologic events that cause a preterm delivery (PTD) is not completely understood till date. Genetic predisposition, maternal risk factors (e.g., age, smoking, alcohol intake, reproductive history), urinary tract infections, intrauterine infections, etc., are typically associated with increased PTD risk. Given the variety of predisposing factors that contribute to an increased PTD risk^6,7^, there exist intervention approaches that can potentially promote a healthy full-term gestation outcome^8,9^. However, the success of these intervention approaches is dependent on identification of high-risk subjects as early as possible during pregnancy^3^. Given this context, diagnostic markers (physical and/or biochemical) that can accurately indicate, at an early stage of pregnancy, the possibility of progression towards a preterm delivery outcome assume a lot of significance^10,11^. Figure 1 provides an overview of approaches (and few commercial *in vitro* diagnostic offerings) used for determining PTD risk. Given below is a summary of various methods depicted in Fig. 1.

Briefly, general factors considered for ‘risk scoring’ include ethnicity, socio-economic status, periodontal health, blood pressure, weight, diabetes, smoking, alcohol consumption, inter-pregnancy duration, etc.^10,12–14^. The clinical predictive values of the stated factors are however quite limited. Pathologic manifestations such as bacterial vaginosis or urinary tract infections during pregnancy, unless properly addressed, are also known to adversely impact pregnancy outcome in most cases^15–18^. Several studies have also investigated associations between ‘physical markers’ (e.g. cervical length, uterine artery pulsatility index, etc.) and the eventual pregnancy outcome (term or preterm)^19,20^. Most studies indicate cervical length shortening in the second trimester of pregnancy to be associated with higher risk of spontaneous preterm birth^19,21–23^. More than its utility in indicating an impending preterm birth outcome, cervical length appears to have a good negative predictive value. CerviLenz is a commercially available device that finds utility in measurement of cervical length^24^.

A few biochemical markers identified from cervico-vaginal secretions, amniotic fluid, urine, saliva, serum, and plasma also find utility in assessment of premature delivery risk (Fig. 1). These include inflammation markers like cervical IL-6, serum C-reactive protein (CRP), and other proteins like fetal fibronectin, β-hCG, placental α-microblobulin etc.^10,25–30^. Amongst these, fetal fibronectin (Ffn) has been reported to be the most effective marker, having more than 60% sensitivity in predicting spontaneous preterm births, based on sampling done during ~22–24 weeks of gestation^27,28^. Inflammation markers like IL-6, tested at a similar time-period during pregnancy, can also predict an impending preterm delivery, albeit with lesser sensitivity^10,31^. On the other hand, hormonal markers like β-hCG have been reported to predict spontaneous preterm birth outcomes with high sensitivity, but are applicable only during late stages of pregnancy (~34 weeks of gestation)^32,33^. Some recently developed *in vitro* diagnostic tests rely on comprehensive proteomic and metabolomic analyses of biological samples (blood, amniotic fluid, etc.) and combine multiple risk predictors in order to increase sensitivity of prediction. For example, while a test offered by SERA prognostics provides risk assessment based on the detected levels of two blood proteins (viz. SHBG & IBP4)^34,35^, another test offered by Metabolon relies on a metabolomic analysis of the amniotic fluid^36^. It is likely that high costs and limited accuracy (~60–80%) of the methods depicted in Fig. 1 deter gynaecologists and public health organisations from recommending routine (wide-spread) clinical usage. The false positive prediction rates of depicted methods also remain high^10,11^. Even a single false prediction will needlessly subject a pregnant woman to unnecessary mental turmoil, whilst also incurring personal/state-funded diagnostic and monitoring costs.

In a significant shift from physical and/or biochemical diagnostic markers, a few recent studies have indicated the potential of employing characteristics of vaginal microbial communities (in pregnant women) as a diagnostic marker for predicting pre/full term outcomes^23,37–44^. Observations indicate that taxonomic profiles derived from vaginal microbiomes of preterm subjects tend to cluster as a somewhat distinct group (typically referred to as CST viz., community state type). The consistent presence of species belonging to known bacterial pathogens such as *Gardenerella*, *Atopobium*, *Ureaplasma*, etc., (with certain abundance) in samples grouping into a preterm delivery associated CST, has fuelled a lot of research focus in this direction^37^. Reports from these efforts also indicate subtle differences in alpha-diversity metrics (particularly with respect to species diversity and evenness measures) between taxonomic profiles obtained from vaginal microbiome samples taken from preterm and full-term subjects^38,40^.

In this study, we performed a systematic analysis of taxonomic diversity profiles corresponding to 1621 publicly available vaginal microbiomes (sampled from 303 pregnant women) pooled from four recent studies^37,38,44,45^. Table 1 provides details of the four studies. The aim was to understand and investigate temporal differences, if any, between the community structures of vaginal microbiomes sampled from pregnant women during various stages of their pregnancy. Further, suitable experiments were designed for evaluating and comparing the efficiency of various diversity measures in differentiating between vaginal microbiomes sampled from pregnant women with reported “term” or “preterm” delivery outcomes. The overall objective of this study was to obtain a possible answer for the following question. Are there potential (temporal) signatures in the microbial community structure of vaginal samples (in pregnant women) that can indicate predisposition to preterm birth?

**Table 1:** Details of microbiome studies considered in the present analysis.

Study (No. of subjects)	Reference	No. of term samples	No. of preterm samples	16S variable region amplified for sequencing
Study 1 (37)	DiGiulio *et al*., PNAS, Sep 2015 (doi:10.1073/pnas.1502875112)	517	181	V3–V5
Study 2 (41)	Romero *et al*., Microbiome, May 2014 (doi:10.1186/2049-2618-2-18)	163	43	V1–V3
Study 3 (22)	Romero *et al*., Microbiome, Feb 2014 (doi:10.1186/2049-2618-2-4)	139	—	V1–V3
Study 4 (173)	https://www.ncbi.nlm.nih.gov/bioproject/?term=prjeb10913 (Project id: PRJEB10913)	578	—	V4–V5

Only those microbiome samples were considered that had at least 500 taxonomically assigned sequences and were collected from pregnant women within 40 weeks of gestation.

## Results and Discussion

**Figure 2:**
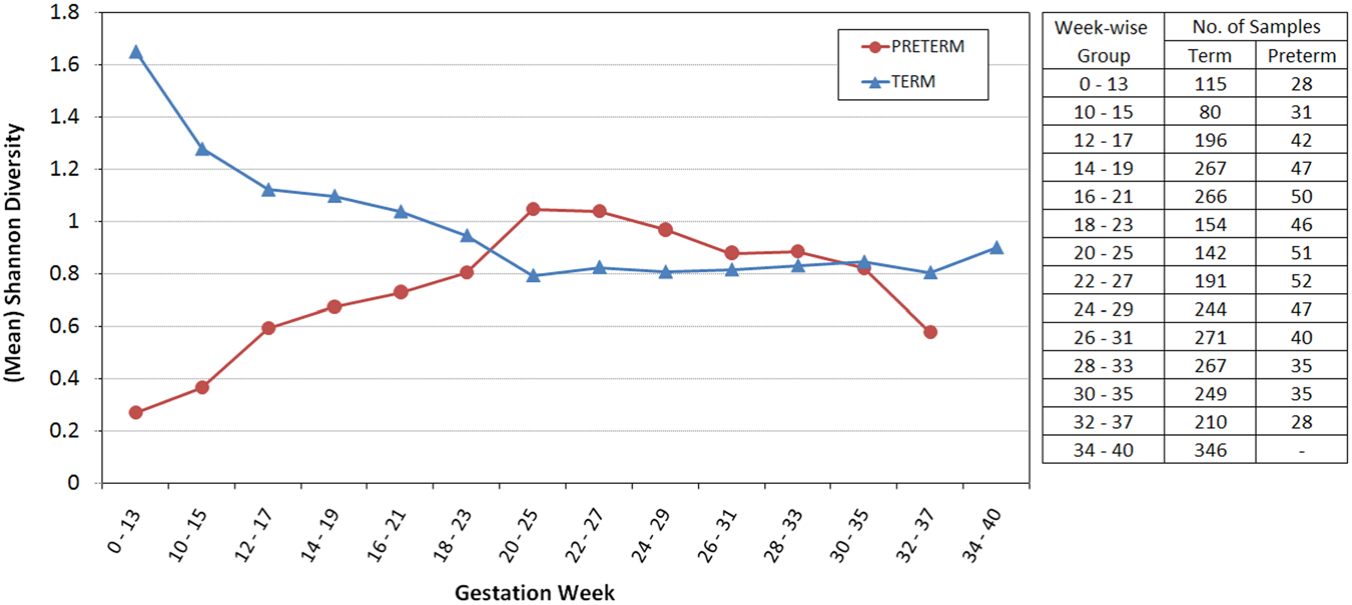
Shannon diversity trends across the 15 temporally overlapping groups for the evaluated ‘term’ and ‘preterm’ samples. Results indicate that women with preterm delivery outcomes tend to have lesser diversity in their vaginal microbiome during their first 15–20 weeks of pregnancy as compared to women with term delivery outcomes.

Changes in vaginal microbiome diversity across various stages of pregnancy were first evaluated using Shannon diversity^40,46^ as a metric. In order to obtain a clear picture of microbial community transition at various time points in pregnancy, and to minimize the effects of outliers, taxonomic profiles corresponding to 1621 samples pooled from 4 studies (Table 1) were divided into 15 overlapping week-wise groups. Shannon diversity values of each of the samples were computed using their respective taxonomic abundance profiles. Figure 2 depicts the Shannon diversity trends across the 15 temporally overlapping groups for the evaluated ‘term’ and ‘preterm’ samples. Results indicate that women with preterm delivery outcomes tend to have lesser diversity in their vaginal microbiome during their first 15–20 weeks of pregnancy as compared to women with term delivery outcomes. After approximately 20 weeks of pregnancy, the vaginal microbiome diversity in case of both term and preterm outcomes appear to converge and remain more or less stable in the remaining weeks of pregnancy.

The observed temporal differences (with respect to Shannon diversity) between microbial communities in vaginal samples taken from subjects with term or preterm delivery outcomes indicate the possibility of employing a suitable diversity metric that can effectively capture the microbial community structure (in early weeks of pregnancy) and can therefore be potentially employed as a screening/diagnostic marker for predicting preterm delivery risk. Given this context, the following experiments were performed with the objective of evaluating and comparing the capability of various existing diversity measures (including Shannon diversity) in predicting pregnancy delivery outcomes (term or preterm) from a taxonomic profile corresponding to a sampled vaginal microbiome.

All available microbiomes were segregated into 33 week-wise groups. Group ‘Week_N_’ comprised of all vaginal microbiomes that were sampled at any time-point on or before the N^th^ week of pregnancy (N ranging between 8–40). Microbiomes in each group were labelled into classes ‘Term’ or ‘PTD’, denoting (reported) ‘full-term’ or ‘preterm’ delivery outcome respectively. Besides Shannon diversity, other widely used/reported alpha diversity indices, viz., Chao1 and Simpson, denoting species richness and evenness respectively^47–49^, were computed for taxonomic profiles corresponding to microbiomes in all groups. Given that vaginal microbiomes have an overwhelming predominance of microbial species belonging to *Lactobacillus*, with other taxa playing the minority role (as the ‘tail’), microbial abundance distribution (upon ordering) resembles a highly skewed Lorenz curve (analogous to inequitable distribution of wealth in human populations). Keeping this in mind, statistical measures of inequality, viz., Gini, Atkinson, Theil, Decile ratios, and Ricci-Schutz indices were also computed for individual taxonomic profiles of all vaginal microbiomes^50–55^. For each group, the diagnostic value (ability) of individual indices to differentiate between term and PTD outcomes was estimated in terms of Mathews Correlation Coefficient (MCC), a measure that captures both specificity and sensitivity (details in methods section), at a threshold value that best separates the compared classes^56^. ‘Extent of Segregation’ (ES), an additional feature (Supplementary Figure 1) that quantifies the differentiating capability of a metric was also computed in order to evaluate metrics achieving a perfect MCC value of 1 (i.e. complete separation between case and control samples). In addition to the mentioned diversity and inequality metrics, we also computed ‘Taxonomic Composition Skew’ (TCS), a novel metric, for all samples. Supplementary Information provides a brief background that places TCS metric in the context of other existing diversity and inequality metrics, and the methodology adopted for computing TCS (along with a worked-out example).

Results depicted in Table 2 indicate that all evaluated diversity and inequality measures obtain positive MCC values. This clearly indicates significant differences (in taxonomic diversity) between vaginal microbiome samples obtained from women with term or PTD outcomes. Interestingly, these differences are observed to be more pronounced in (approximately) the first trimester of pregnancy (<15 gestation weeks). With increasing gestational age, the MCC values for various indices progressively decrease, but still remain above a value of 0.25. Although this indicates that diversity and inequality measures (in the late second and third trimesters of pregnancy) exhibit contrasting trends between term and PTD cases at a population level, their utility in subject-specific risk assessment appears to be limited. Amongst the compared indices, TCS is observed to outperform others, with the difference appearing more prominent until 15–20 weeks of gestation (Table 2). A statistical comparison (employing Wilcoxon signed rank test) indicates that MCC values obtained using TCS (until 20^th^ week of pregnancy) are significantly higher (Benjamini-Hochberg corrected p-values < 0.008) than that obtained using other diversity and inequality measures. Furthermore, TCS is observed to obtain perfect MCC values of 1 (until Week-10), thereby indicating that a vaginal microbiome sample (obtained from a subject at any time point in pregnancy on or before the 10^th^ gestation week) can be employed for accurately diagnosing/predicting the risk of a preterm delivery outcome while avoiding false alarms. Even when samples obtained on or before 20^th^ gestation week were considered, TCS could provide an MCC value of 0.823, indicating sufficiently high sensitivity and specificity of prediction. A comparison of ‘Extent of Segregation’ (ES) values obtained for various metrics also indicates that ES provided by TCS is significantly higher (Wilcoxon signed rank test, Benjamini-Hochberg corrected p-values < 0.005) than that obtained with other metrics. Higher ES values indicate increased confidence with respect to the differentiating capability of the metric.

**Table 2:** Capability of various diversity indices and inequality measures in differentiating between vaginal microbiome samples corresponding to term and preterm delivery outcomes.

Gestation weeks	Number of evaluated microbiome samples			Mathews Correlation Coefficient (MCC)* obtained with various diversity and inequality measures								
(Week_N_)	PTD	TERM	Total	TCS	Shannon	Simpson	Chao1	Gini	Ricci-Schutz	Atkinson	Theil	Decile ratio (90:10)
8	6	64	70	**1** (25.65)	1 (7.59)	1 (8.64)	1 (7.51)	1 (0.90)	0.804	0.578	0.818	0.560
9	7	70	77	**1** (21.84)	0.929	0.929	1 (4.51)	0.929	0.833	0.637	0.833	0.637
10	10	77	87	**1** (21.84)	0.887	0.887	1 (4.51)	0.947	0.755	0.622	0.839	0.556
11	13	86	99	**0.958**	0.872	0.823	0.958	0.920	0.763	0.701	0.864	0.593
12	19	97	116	**0.937**	0.840	0.805	0.937	0.874	0.731	0.673	0.811	0.615
13	28	115	143	**0.956**	0.842	0.701	0.933	0.843	0.744	0.701	0.817	0.613
14	35	135	170	**0.947**	0.849	0.676	0.928	0.853	0.774	0.713	0.835	0.633
15	41	157	198	**0.940**	0.849	0.672	0.923	0.843	0.775	0.687	0.842	0.600
16	51	200	251	**0.888**	0.758	0.592	0.868	0.849	0.741	0.712	0.836	0.598
17	61	293	354	**0.835**	0.606	0.481	0.822	0.835	0.729	0.705	0.813	0.550
18	72	364	436	**0.829**	0.576	0.469	0.820	0.808	0.717	0.699	0.792	0.528
19	82	402	484	**0.842**	0.572	0.470	0.826	0.808	0.702	0.680	0.793	0.514
20	89	445	534	**0.823**	0.545	0.437	0.807	0.794	0.688	0.668	0.781	0.491
21	101	466	567	**0.751**	0.500	0.423	0.732	0.722	0.624	0.603	0.710	0.446
22	109	493	602	**0.719**	0.452	0.369	0.702	0.704	0.598	0.587	0.686	0.408
23	118	518	636	**0.668**	0.422	0.338	0.658	0.648	0.544	0.534	0.621	0.372
24	129	554	683	**0.629**	0.396	0.313	0.620	0.618	0.511	0.498	0.580	0.354
25	140	587	727	0.588	0.353	0.302	0.574	**0.590**	0.498	0.492	0.552	0.330
26	150	637	787	0.560	0.333	0.301	0.554	**0.566**	0.474	0.460	0.527	0.308
27	161	684	845	0.522	0.301	0.294	0.523	**0.542**	0.463	0.450	0.515	0.307
28	170	741	911	0.505	0.310	0.304	0.502	**0.524**	0.445	0.419	0.490	0.299
29	176	798	974	0.485	0.302	0.299	0.488	**0.519**	0.445	0.415	0.489	0.293
30	184	850	1034	0.463	0.292	0.290	0.465	**0.490**	0.418	0.400	0.468	0.289
31	190	908	1098	0.453	0.298	0.299	0.457	**0.487**	0.413	0.391	0.463	0.281
32	196	962	1158	0.436	0.301	0.302	0.442	**0.471**	0.388	0.367	0.442	0.273
33	205	1008	1213	0.431	0.310	0.310	0.433	**0.448**	0.370	0.354	0.423	0.276
34	213	1051	1264	0.427	0.321	0.317	0.428	**0.434**	0.359	0.347	0.408	0.273
35	219	1099	1318	0.414	0.324	0.320	0.417	**0.427**	0.355	0.342	0.401	0.272
36	222	1137	1359	0.402	0.317	0.315	0.407	**0.418**	0.346	0.341	0.390	0.273
37	224	1172	1396	0.391	0.322	0.320	0.395	**0.408**	0.339	0.331	0.382	0.271
38	224	1333	1557	0.392	0.324	0.321	0.396	**0.407**	0.343	0.331	0.382	0.266
39	224	1376	1600	0.388	0.322	0.319	0.391	**0.403**	0.339	0.330	0.377	0.264
40	224	1397	1621	0.383	0.323	0.319	0.388	**0.397**	0.334	0.328	0.371	0.265

*At each gestation week, the best attained MCC value is indicated in bold face font. A higher MCC value indicates that the metric is able to better differentiate between microbiome samples corresponding to term and preterm delivery outcomes. In cases where a threshold diversity/inequality value could completely differentiate/segregate between samples corresponding to term and preterm delivery outcomes (i.e. MCC = +1), the extent of segregation (ES) is indicated in brackets. A higher ES value indicates relatively better separation between the two groups of samples (term and preterm).

Results obtained using microbiome datasets cumulated from all 4 studies indicate existence of differences in vaginal microbial community structures (during the first 15–20 weeks of pregnancy) in pregnant subjects with term and preterm delivery outcomes. However, with increasing gestation age, these (diversity) differences are observed to become less pronounced. In order to assess whether these differences in term and preterm vaginal microbial communities (in the “early” stages of pregnancy) remain consistent between individual studies and across datasets sampled from women with diverse ethnicities, suitable internal and external cross-validation experiments were designed and performed in the following manner.

Internal cross-validation experiments were performed using vaginal microbiome taxonomic profiles corresponding to two sets of samples belonging to study 1, which was the largest amongst the four studies in terms of number of samples (Table 1). The two sets comprised of samples that were obtained during the first 15 weeks (set 1) and the first 20 weeks (set 2) of pregnancy, respectively. In each set, two-thirds of samples were randomly selected and used as a ‘training corpus’ for determining an optimal threshold value of TCS metric that would provide maximal separation (quantified in terms of MCC) between the microbiome samples corresponding to preterm (case) delivery and term (control) delivery outcomes. The efficiency of the determined “threshold” value (in predicting pregnancy outcome) was subsequently evaluated using the corresponding ‘test corpus’, which comprised of data pertaining to the remaining one-third of the microbiome samples. In each set, the above cross validation procedure was iterated 1000 times. During each of these iterations, the ability of the determined “threshold” TCS value to differentiate between microbiome samples (of respective test corpus) corresponding to cases (preterm) and controls (term) was evaluated in terms of six parameters, namely, accuracy, sensitivity, specificity, positive predictive value (PPV), negative predictive value (NPV), and Matthews’s correlation coefficient (MCC). For the purpose of comparison, all other diversity and inequality metrics were also subjected to the same internal cross-validation exercise.

Results, in terms of six evaluation parameters (mentioned above), obtained across 1000 iterations of testing are summarized in Tables 3 and 4. Mean values of the evaluation parameters (along with respective standard deviations) generated from set 1, as well as, set 2 are depicted in Tables 3 and 4, respectively. Results in these tables are observed to concur with earlier observations depicted in Table 2. Diversity of microbial communities indeed appears to be a good indicator of pregnancy outcome. Amongst the compared metrics, TCS is observed to have better prediction efficiency and a good balance between specificity and sensitivity of prediction (which is also reflected in the MCC values). A statistical comparison of evaluation parameter values obtained (across 1000 iterations) using TCS metric with those obtained using other metrics for both subsets of (testing corpus) samples are further provided in Tables 3 and 4 respectively. As evident from the data shown in these tables, in majority of cases, TCS obtains significantly higher values of accuracy, sensitivity, specificity, PPV, NPV, and MCC, thereby clearly indicating its potential utility in accurately diagnosing/predicting the risk of the preterm delivery from vaginal microbiome samples in the early stages (i.e. first 15–20 weeks) of pregnancy.

**Table 3:** Results of internal cross-validation experiments providing a comparison of the efficiency of the various evaluated diversity and inequality metrics in predicting a preterm delivery outcome.

	Accuracy	Sensitivity	Specificity	PPV	NPV	MCC
**TCS**	**0.956 (0.03)**	**0.949 (0.08)**	0.960 (0.03)	0.919 (0.07)	**0.976 (0.04)**	**0.902 (0.07)**
	*NA*	*NA*	*NA*	*NA*	*NA*	*NA*
**Shannon**	0.921 (0.04)	0.896 (0.09)	0.934 (0.04)	0.865 (0.09)	0.950 (0.04)	0.821 (0.09)
	*2.07E-94*	1*.79E-45*	1.16*E-65*	*1.22E-83*	*2.2E-58*	*1.7E-99*
**Simpson**	0.849 (0.06)	0.646 (0.13)	0.944 (0.05)	0.851 (0.11)	0.851 (0.06)	0.642 (0.12)
	*4.2E-161*	*7.0E-160*	*5.43E-17*	*6.51E-68*	*5.4E-162*	*2.8E-163*
**Chao1**	0.932 (0.03)	0.904 (0.08)	0.946 (0.04)	0.890 (0.08)	0.955 (0.04)	0.847 (0.07)
	*3.1E-97*	*2.2E-51*	*6.66E-33*	*6.35E-50*	*1.5E-69*	*4.2E-98*
**Gini**	0.899 (0.04)	0.766 (0.11)	0.962 (0.05)	0.916 (0.10)	0.899 (0.05)	0.768 (0.09)
	*3.4E-146*	*1.2E-147*	*1*	*1*	*1.8E-151*	*2.3E-153*
**Ricci-Schutz**	0.869 (0.05)	0.738 (0.12)	0.931 (0.05)	0.839 (0.11)	0.884 (0.05)	0.694 (0.11)
	*1.7E-158*	*1.7E-153*	*1.01E-56*	*1.72E-97*	*6.2E-159*	*7.5E-161*
**Atkinson**	0.881 (0.05)	0.761 (0.12)	0.938 (0.04)	0.850 (0.09)	0.894 (0.05)	0.720 (0.11)
	*8.8E-152*	*2.7E-149*	*1.72E-44*	*2.86E-82*	*6.2E-156*	*1.1E-157*
**Theil**	0.892 (0.04)	0.757 (0.12)	0.957 (0.06)	0.907 (0.12)	0.895 (0.05)	0.754 (0.09)
	*3.1E-147*	*8.2E-149*	*0.934440884*	*0.167376*	*3.5E-156*	*2.2E-156*
**Decile ratio (90:10)**	0.802 (0.05)	0.405 (0.12)	**0.987 (0.02)**	**0.944 (0.11)**	0.780 (0.06)	0.527 (0.12)
	*2.9E-164*	*1.7E-164*	*1*	*1*	*1.3E-164*	*1.4E-164*

These experiments were performed using vaginal microbiome samples obtained before 15 weeks of gestation. Mean (and standard deviation) values of the six evaluation parameters obtained across 1000 iterations of cross-validation are provided. Benjamini-Hochberg corrected p-values indicating the relatively better efficiency of the TCS metric as compared to other metrics (Wilcoxon paired rank sum test) are also indicated in italics.

**Table 4:** Results of internal cross-validation experiments providing a comparison of the efficiency of the various evaluated diversity and inequality metrics in predicting a preterm delivery outcome.

	Accuracy	Sensitivity	Specificity	PPV	NPV	MCC
**TCS**	**0.965 (0.02)**	**0.970 (0.03)**	0.964 (0.03)	0.922 (0.05)	**0.986 (0.01)**	**0.921 (0.04)**
	*NA*	*NA*	*NA*	*NA*	*NA*	*NA*
**Shannon**	0.855 (0.03)	0.759 (0.10)	0.899 (0.05)	0.773 (0.09)	0.896 (0.04)	0.662 (0.07)
	*2.5E-165*	*5.9E-163*	*1.3E-150*	*3.3E-163*	*2.2E-164*	*2.7E-165*
**Simpson**	0.819 (0.04)	0.489 (0.09)	0.963 (0.02)	0.855 (0.08)	0.812 (0.04)	0.547 (0.08)
	*2.5E-165*	*6.5E-165*	*2.0E-01*	*5.4E-84*	*4.8E-165*	*2.7E-165*
**Chao1**	0.947 (0.02)	0.936 (0.05)	0.952 (0.03)	0.896 (0.05)	0.972 (0.02)	0.878 (0.04)
	*6.1E-129*	*4.7E-81*	*4.8E-49*	*2.0E-78*	*3.3E-117*	*1.4E-138*
**Gini**	0.908 (0.02)	0.820 (0.09)	0.947 (0.04)	0.880 (0.08)	0.925 (0.04)	0.784 (0.05)
	*2.5E-163*	*1.8E-154*	*7.2E-33*	*1.1E-55*	*4.6E-160*	*8.0E-165*
**Ricci-Schutz**	0.868 (0.03)	0.730 (0.09)	0.929 (0.04)	0.826 (0.09)	0.888 (0.04)	0.684 (0.07)
	*8.1E-165*	*6.9E-165*	*2.9E-88*	*5.6E-134*	*4.8E-165*	*2.7E-165*
**Atkinson**	0.859 (0.03)	0.703 (0.11)	0.929 (0.04)	0.820 (0.09)	0.878 (0.04)	0.662 (0.07)
	*2.5E-165*	*6.9E-165*	*1.7E-88*	*1.0E-130*	*4.8E-165*	*2.7E-165*
**Theil**	0.899 (0.03)	0.809 (0.10)	0.940 (0.05)	0.867 (0.09)	0.919 (0.04)	0.766 (0.06)
	*2.3E-163*	*1.0E-157*	*9.0E-48*	*5.1E-65*	*7.1E-162*	*4.3E-165*
**Decile ratio (90:10)**	0.795 (0.04)	0.347 (0.08)	**0.991 (0.01)**	**0.949 (0.08)**	0.777 (0.04)	0.492 (0.07)
	*2.5E-165*	*6.5E-165*	*1*	*1*	*4.8E-165*	*2.7E-165*

These experiments were performed using vaginal microbiome samples obtained before 20 weeks of gestation. Mean (and standard deviation) values of the six evaluation parameters obtained across 1000 iterations of cross-validation are provided. Benjamini-Hochberg corrected p-values indicating the relatively better efficiency of the TCS metric as compared to other metrics (Wilcoxon paired rank sum test) are also indicated in italics.

To further evaluate the robustness of diversity metrics (with respect to their differentiating capability between term and preterm pregnancy outcomes) across studies/ethnicities, the following external validation experiment was performed. Mean thresholds of various metrics (including TCS) obtained during the internal cross-validation experiments (performed using data from Study 1) were employed to check their applicability with microbiome data collected from the other three studies, viz. Studies 2, 3, and 4 (considering them as external validation data). In the current scenario, external validation data should ideally comprise of vaginal microbiome samples taken from pregnant women from a different geography or ethnicity. Although, studies 2 and 3 comprise of samples obtained from American women (similar to that of Study 1), data from these studies have differences with respect to racial distribution of the subject cohort, experimental protocols, and the 16 S variable regions sequenced^37,44^. In an absolute contrast, the external validation data also comprises data from Study 4, wherein samples were obtained from Chinese subjects. Results of external validation are provided in Tables 5 and 6. Similar to the internal cross-validation experiments, external validation was also performed for two subsets of samples using data cumulated till 15 weeks and 20 weeks, respectively. Results, with respect to all six evaluation parameters (viz. accuracy, sensitivity, specificity, PPV, NPV, and MCC), clearly indicate and lend support to the hypothesis regarding distinct differences between vaginal microbial community structure (in early stages of pregnancy) between women with term and preterm delivery outcomes. More so, this hypothesis appears to hold true irrespective of the ethnicity/geography. Results also confirm the relatively better efficiency of TCS metric (compared to other diversity metrics) in capturing the community level differences prevalent in vaginal microbiomes (in the first 15–20 weeks of pregnancy).

**Table 5:** Results of external validation experiments providing a comparison of the efficiency of the various evaluated diversity and inequality metrics in predicting a preterm delivery outcome.

	TCS	Shannon	Simpson	Chao1	Gini	Ricci-Schutz	Atkinson	Theil	Decile ratio (90:10)
**Accuracy**	1.000	0.954	0.931	0.977	0.977	0.954	0.828	0.977	0.966
**Sensitivity**	1.000	0.667	0.333	1.000	0.667	0.667	0.667	0.667	0.500
**Specificity**	1.000	0.975	0.975	0.975	1.000	0.975	0.840	1.000	1.000
**PPV**	1.000	0.667	0.500	0.750	1.000	0.667	0.235	1.000	1.000
**NPV**	1.000	0.975	0.952	1.000	0.976	0.975	0.971	0.976	0.964
**MCC**	1.000	0.642	0.373	0.855	0.807	0.642	0.323	0.807	0.694

These experiments were performed using vaginal microbiome samples obtained before 15 weeks of gestation (from studies 2, 3, and 4).

**Table 6:** Results of external validation experiments providing a comparison of the efficiency of the various evaluated diversity and inequality metrics in predicting a preterm delivery outcome.

	TCS	Shannon	Simpson	Chao1	Gini	Ricci-Schutz	Atkinson	Theil	Decile ratio (90:10)
**Accuracy**	0.924	0.784	0.880	0.897	0.947	0.940	0.937	0.940	0.924
**Sensitivity**	0.667	0.500	0.111	0.778	0.556	0.556	0.611	0.611	0.167
**Specificity**	0.940	0.802	0.929	0.905	0.972	0.965	0.958	0.961	0.972
**PPV**	0.414	0.138	0.091	0.341	0.556	0.500	0.478	0.500	0.273
**NPV**	0.978	0.962	0.943	0.985	0.972	0.972	0.975	0.975	0.948
**MCC**	0.487	0.174	0.037	0.472	0.527	0.495	0.508	0.521	0.175

These experiments were performed using vaginal microbiome samples obtained before 20 weeks of gestation (from studies 2, 3, and 4).

It is pertinent to mention here that the number of microbiome samples corresponding to preterm delivery outcome that could be collated for this study were limited. Availability of data from other ethnicities and geographies (besides US and China) would be essential for a more robust statistical validation of the above findings. It is expected that more data will become available in the coming years as interest in this field grows, allowing for a more comprehensive and statistically revealing meta-analysis. Furthermore, the concept of microbiome based diagnostics is still in its infancy, and translating the findings of this study to a clinical setting will have its own set of challenges.

## Conclusion

The capability to ‘accurately’ predict a preterm delivery outcome, right in the first trimester of pregnancy, enables the following. Primarily, early prediction allows application/administration of available physical and/or pharmacological interventions (either prophylactic or therapeutic) to the concerned subject, with an aim of reducing/completely obviating the impending risk. Moreover, it helps in initiating monitoring/surveillance of the concerned subject and suitably enhancing levels of antenatal care. On a different note, early and (more importantly) ‘accurate’ prediction also finds application in identifying/recruiting a cohort of high-risk subjects willing to participate in clinical trials of novel intervention techniques that reduce the risk of preterm delivery outcomes and associated complications^57^.

In summary, this work explores and validates the utility of vaginal microbiome diversity in enabling ‘early’ prediction of preterm delivery outcomes. This work also introduces a novel diversity metric (TCS) that can accurately predict a preterm delivery outcome, as early as in the first trimester of pregnancy. Validation results indicate the potential utility of employing TCS metric in a clinical diagnostic setting (for accurate preterm birth risk assessment). We anticipate that the presented findings have far reaching implications in the fight against neonate mortality resulting due to preterm births.

## Methods

OTU level taxonomic profiles (Greengenes OTUs version 13.5, clustered at 97% identity) corresponding to vaginal microbiome samples from four studies^37,38,44,45^ were obtained. Alpha diversity (Shannon, Simpson, Chao1) and inequality measures (Gini-coefficient, Ricci-Schutz, Atkinson, Theil, and Decile ratio) corresponding to the taxonomic profiles were calculated using R packages vegan (v2.0–10) and ineq (v0.2–13), respectively. In addition, the skew in abundances of different microbial groups in the taxonomic profiles was computed using the novel metric – TCS (details in Supplementary information). Evaluation parameters, viz. MCC, AUC, specificity, sensitivity, accuracy, PPV and NPV were calculated using R packages – ROCR, cross val v.1.0.3, and pROC. Matthews Correlation Coefficient (MCC), a measure that captures both specificity and sensitivity of prediction/classification using a selected threshold value of the index under consideration, was computed using the equation below.

1 $${\rm{MCC}}=\frac{{\rm{TP}}\times {\rm{TN}}-{\rm{FP}}\times {\rm{FN}}}{\sqrt{({\rm{TP}}+{\rm{FP}})({\rm{TP}}+{\rm{FN}})({\rm{TN}}+{\rm{FP}})({\rm{TN}}+{\rm{FN}})}}$$

Wherein, TP, TN, FP, and FN represent the number of true-positive predictions, true-negative predictions, false-positive predictions, and false-negative predictions, respectively. A perfect MCC value of +1 indicates complete separation between the microbiome samples corresponding to the preterm delivery and the term delivery. Other evaluation parameters were calculated from the generated confusion matrices using the following formulae (equations 2–6):

2 $${\rm{Accuracy}}=({\rm{TP}}+{\rm{TN}})/({\rm{TP}}+{\rm{TN}}+{\rm{FP}}+\text{FN})$$

3 $${\rm{Sensitivity}}={\rm{TP}}/({\rm{TP}}+{\rm{FN}})$$

4 $${\rm{Specificity}}={\rm{TN}}/({\rm{TN}}+{\rm{FP}})$$

5 $${\rm{PPV}}={\rm{TP}}/({\rm{TP}}+{\rm{FP}})$$

6 $${\rm{NPV}}={\rm{TN}}/({\rm{TN}}+{\rm{FN}})$$

The extent of segregation (ES), an additional feature that quantifies the differentiating capability of various metrics evaluated in the present study was computed using the following equation.

7 $${\rm{Extent}}\,{\rm{of}}\,\text{Segregation}\,({\rm{ES}})=\frac{\min [{\rm{\delta }}(\max \,{{\rm{D}}}_{{\rm{TD}}},\,{{\rm{minD}}}_{{\rm{PTD}}}),\,{\rm{\delta }}\,(\max \,{{\rm{D}}}_{{\rm{PTD}}},\,{{\rm{minD}}}_{{\rm{TD}}})]}{\max \,[{\rm{\delta }}(\max \,{{\rm{D}}}_{{\rm{TD}}},\,{{\rm{minD}}}_{{\rm{PTD}}}),\,{\rm{\delta }}\,(\max \,{{\rm{D}}}_{{\rm{PTD}}},\,{{\rm{minD}}}_{{\rm{TD}}})]}\,\times \,100$$

Wherein,

D_TD_→ set of values calculated for a given diversity/inequality metric for all samples corresponding to term delivery outcomes.

D_PTD_→ set of values calculated for a given diversity/inequality metric for all samples corresponding to preterm delivery outcomes.

δ (max D_TD_, min D_PTD_) → absolute difference between the maximum value of the set D_TD_ and the minimum value of the set D_PTD_.

δ (max D_PTD_, min D_TD_) → absolute difference between the maximum value of the set D_PTD_ and the minimum value of the set D_TD_.

**Figure 3:**
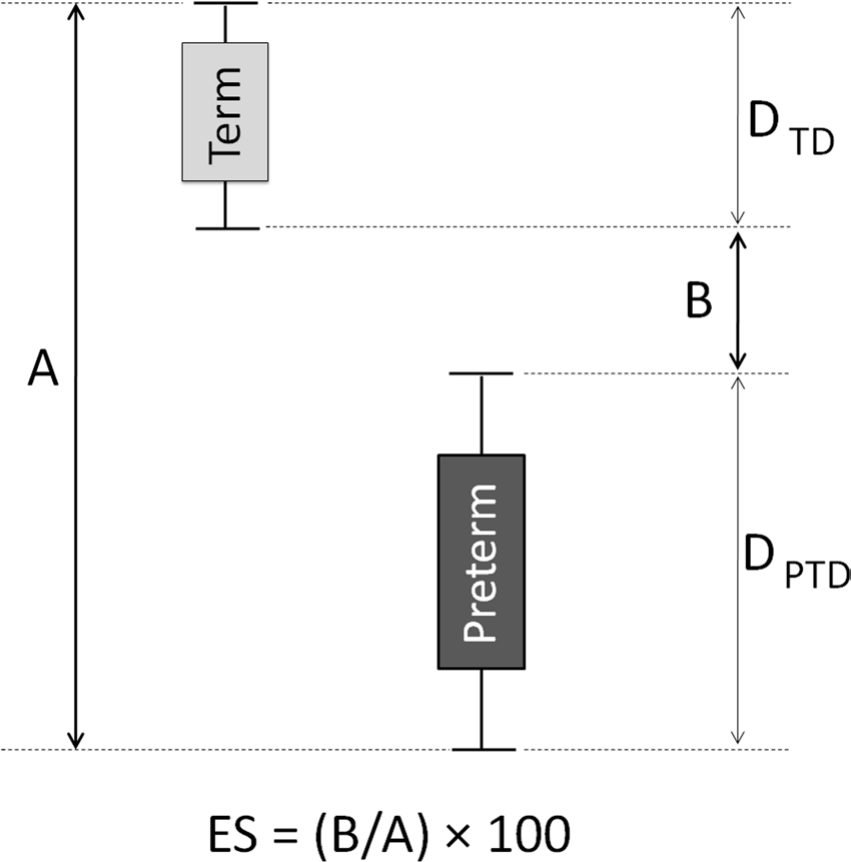
Example depicting calculation of extent of segregation (ES), indicating how ES captures the discriminating ability of a metric used for binary classification of microbiome samples. A higher ES value indicates a better separation between the two groups of samples.

A higher ES value indicates a better separation between the two groups of microbiome samples. Figure 3 diagrammatically depicts an example of calculation of extent of segregation (ES), and indicates how ES captures the discriminating ability of an ecological-diversity or an economic-inequality metric.

## Electronic supplementary material

Supplementary Information
