# Supplementary material for: First-trimester vaginal microbiome diversity: A potential indicator of preterm delivery risk
Source: Sci Rep. 2017 Nov 23;7:16145. doi: 10.1038/s41598-017-16352-y (PMC5700938; doi:10.1038/s41598-017-16352-y)
Supplement: Supplementary file 1 — Supplementary Information [file 41598_2017_16352_MOESM1_ESM.pdf]

Supplementary Information

for

First-trimester vaginal microbiome diversity: A definitive indicator of preterm delivery risk

Mohammed Monzoorul Haque[1], Mitali Merchant[1], Pinna Nishal Kumar[1], Anirban Dutta[1], and Sharmila S. Mande[1]

1. Bio-Sciences R&D Division, TCS Research, Tata Consultancy Services Ltd., Pune, India

Supplementary Information

Taxonomic Composition Skew (TCS)

The following sections provide

1. **Background** about TCS metric
2. **Steps** followed for computing TCS
3. A **worked-out example** depicting the methodology for computing TCS

BACKGROUND

Vaginal microbiomes have an overwhelming predominance of microbial species belonging to *Lactobacillus*, with other taxa playing the minority role (forming the 'tail' of such an 'uneven' distribution). Such a microbial abundance distribution (upon ordering) resembles a highly skewed Lorenz curve (analogous to inequitable distribution of wealth in human populations). Therefore, statistical measures of inequality such as Gini Index, Decile ratios, Atkinson, Ricci-Schultz, Theil, etc., can, in principle be utilized for performing direct and objective comparisons (of diversity or inequality) between different microbial abundance distributions[1].

Gini coefficient, one of the most widely cited measure of inequality, computes the ratio of the area between the Lorenz curve and the 45-degree line to the area beneath the 45-degree line[2,3]. In a perfectly equal society, wherein all stakeholders share identical incomes, the Lorenz curve follows the line of equality (i.e. the 45-degree line), thus resulting in a Gini coefficient value of 0. Greater the Lorenz curve deviates from the line of equality, higher is the resulting value of the Gini coefficient. Despite the utility of Gini Index in quantifying inequalities in wealth distribution of a population, it has a few known limitations. Two differing patterns of income distribution (manifested as intersecting Lorenz curves) may result in very similar (or even identical) Gini coefficient values[4]. Furthermore, response of Gini coefficient to income variations between sections of people at the two extremes of the Lorenz curve is not as pronounced as compared to that corresponding to sections of population in the middle of the distribution[4]. Given the analogous relationship between incomes and microbial abundances, employing Gini coefficient as a measure of skew in microbial abundances is also fraught with similar limitations.

'Decile ratios' based metric is another popular metric for studying Lorenz curves (depicting unequal distribution of wealth)[9].These ratios express the income (or income share) of the rich as a multiple of those of the poor. Specific decile ratios (D90:D10 or D80:D20 or D70:D30, etc.,) are employed for quantifying (as a ratio) the abundance skew in wealth accumulated by richer sections of society as compared to the rest to the population. Although a fair estimate of inequalities in wealth distribution is provided, this methodology becomes vulnerable in the presence of outliers with extreme values. Moreover, employing a single decile ratio may not be appropriate for studying all types of distributions and for drawing conclusions regarding the skew in income distributions. Other popular inequality indices (Atkinson, Ricci-Schultz, Theil, etc.,) also face similar issues with respect to their applicability to varied (and highly skewed) distribution patterns[1].

Drawing inspiration from the above metrics (and keeping their limitations in view), we designed a novel metric called 'Taxonomic Composition Skew' (TCS), which represents an overall (median) measure of skews in abundance of different taxonomic groups present in a microbial community. TCS responds differently to changes in abundance patterns along/ across different sections of the microbial community (either the dominant taxa or the sparse taxa) and provides insights into the unequal distribution of taxonomic abundance. By performing a rigorous/ exhaustive comparison of abundance patterns of various taxonomic groups (in a given distribution), the TCS metric potentially enables sensitivity analyses that quantifies the importance of different sections of the income spectrum (analogous to dominant and sparsely abundant taxonomic groups in a microbial community) as determinants of overall wealth inequality (analogous to a skewed or uneven abundance distribution of constituent microbes).

STEPS FOR COMPUTING 'Taxonomic Composition Skew' (TCS)

The method followed for computing 'Taxonomic Composition Skew' (TCS) for a given 'microbial abundance profile' (metagenomic sample) is given below-

Step 1. From a 'microbial abundance profile' corresponding to a microbiome sample, create a list 'L' of various taxonomic groups (present in the sample) wherein individual taxonomic groups (hereafter referred to as 'taxon/ taxa') are ranked in the order of their increasing abundances. The topmost (i.e. top ranked) entries in the list 'L' therefore correspond to 'sparse taxa', and the entries at the end of the list (i.e. bottom ranked) correspond to 'dominant taxa'.

Step 2a. Count the minimum number of 'sparse taxa' (ST_1) whose cumulative abundance is ≥1% of the total population (represented in the microbial abundance profile), when abundance values are progressively cumulated in order of increasing rank of each taxon in list 'L'.

Step 2b. Similar to the step 2a, count the minimum number of 'sparse taxa' (ST_2) whose cumulative abundance is ≥2% of the total population (represented in the microbial abundance profile), when abundance values are progressively cumulated in order of increasing rank of each taxon in list 'L'.

Step 2c. Repeat the step(s) as in 2a & 2b to generate a set 'S' consisting 50 values, representing the counts of minimum number of 'sparse taxa' (ST_i) whose cumulative abundance is ≥ i% (when i = 1, 2, 3, ..., 50) of the total population (represented in the microbial abundance profile), when abundance values are progressively cumulated in order of increasing rank of each taxon in list 'L'.

$S = \{ ST_1 , ST_2 , ST_3 , \ldots\ldots ST_{50} \}$

[**Note**: By definition ST_i are non-zero integers, and $ST_{(i+1)} \geq St_i$]

Step 3a. Count the minimum number of 'dominant taxa' (DT_1) whose cumulative abundance is ≥1% of the total population (represented in the microbial abundance profile), when abundance values are progressively cumulated in order of decreasing rank of each taxon in list 'L'.

Step 3b. Similar to the step 3a, count the minimum number of 'dominant taxa' (DT_2) whose cumulative abundance is ≥2% of the total population (represented in the microbial abundance profile), when abundance values are progressively cumulated in order of decreasing rank of each taxon in list 'L'.

Step 3c. Repeat the step(s) as in 3a & 3b to generate a set 'D' consisting 50 values, representing the counts of minimum number of 'dominant taxa' (DT_j) whose cumulative abundance is ≥ j% (when j = 1, 2, 3, …. 50) of the total population (represented in the microbial abundance profile), when abundance values are progressively cumulated in order of decreasing rank of each taxon in list 'L'.

$D = \{ DT_1 , DT_2 , DT_3 , \ldots\ldots DT_{50} \}$

[**Note**: By definition DT_j are non-zero integers and $DT_{(j+1)} \geq Dt_j$]

Step 4. Compute Cartesian product of the sets 'D' and 'S' to obtain a set 'C' containing 2500 couples (i.e. ordered-pairs) corresponding to DT_j and ST_i values.

$C = D \times S = \{ (DT_j , ST_i) \mid \text{where } DT_j \in D \text{ and } ST_i \in S$ and 'i' and 'j' are positive integers $\leq 50 \}$

Step 5. Derive a set 'DSR' which comprises of values obtained by computing ($DT_j \div ST_i$) for each couple in set 'C'.

$DSR = \{ (DT_j \div St_i) \mid \text{where } (DT_j , ST_i) \in C$ and 'i' and 'j' are positive integers $\leq 50 \}$

Step 6. Calculate 'Taxonomic Composition Skew' (TCS) using the following formula –

TCS = 1 – median (DSR)

Note: In case of a perfectly even distribution of taxonomic groups in a microbiome sample, ($DT_j \div ST_i$) can have a maximum value of 1. Consequently, the TCS index of any microbiome sample can vary in the range: $0 \leq TCS < 1$

The following diagram depicts the calculation of ST and DT values from an ordered (increasing abundance) distribution of taxa present in a metagenomic sample. DT_{25} and ST_{25} values have been calculated as examples.

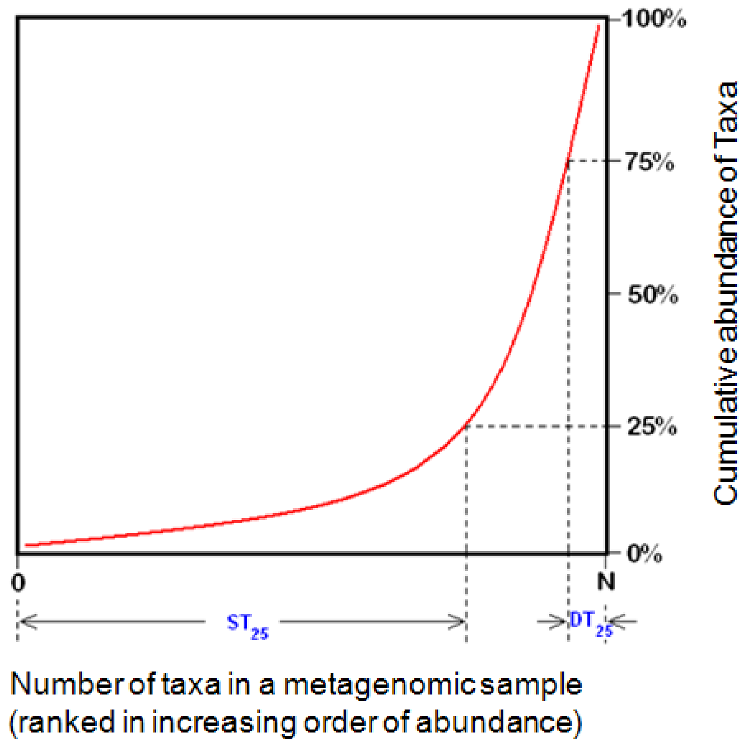
100%
75%
50%
25%
0%
Cumulative abundance of Taxa
0
N
ST_{25}
DT_{25}
Number of taxa in a metagenomic sample
(ranked in increasing order of abundance)

Worked-out example illustrating various steps involved in the computation of 'Taxonomic Composition Skew' (TCS)

The worked-out example illustrated below depict various steps employed for computing the 'Taxonomic Composition Skew' (TCS) metric by analyzing abundance values of various microbes in a given microbiome sample. The said abundance values have been represented in the form of a table (hereafter referred to as 'Microbial Taxonomic Abundance Profile'). Supplementary Table 1 (below) depicts the Microbial Taxonomic Abundance Profiles for a hypothetical microbiome sample (Number of sequences: 49345).

Supplementary Table 1. Microbial taxonomic abundance profile containing abundance values of various microbes (sorted in descending order of their abundance) present in a hypothetical microbiome sample (S1). Below the abundance profile, the value of 'M' i.e. the total population count is also indicated. The value of M is obtained by computing a sum of the abundance values of the plurality of microbes from the microbial taxonomic abundance profile. It may be noted that the numeric value of 'abundance' provided against each Taxon ID (microbe) represents the absolute count of DNA sequences in the sequenced microbiome sample assigned to that Taxon ID by the taxonomic classification technique employed.

**Sample 1 (S1)**	
**Taxon id**	**Abundance**
1	1
2	1
3	1
4	1
5	76
6	241
7	745
8	1027
9	2786
10	3598
11	4863
12	6721
13	7458
14	9852
15	11974
**$M_{(S1)}$ = 49345**	

Starting from a given ‘Microbial Taxonomic Abundance Profile’ (as depicted in Supplementary Table 1), the following steps are involved in the computation of ‘Taxonomic Composition Skew’:

Step 1 Obtaining a total population count 'M' (depicted in Supplementary Table 1) by computing a sum of the abundance values of the plurality of microbes from the microbial taxonomic abundance profile.

Step 2 Creating a sorted list 'L' containing the abundance values of each of the plurality of microbes from the microbial taxonomic abundance profile, wherein the sorted list 'L' comprises of the abundance values of each of the plurality of microbes present in the microbial taxonomic abundance profile ranked in an increasing or a decreasing order of the abundance values. In this one or more top ranked entries in the sorted list ‘L’ correspond to ‘sparse taxa’, and one or more bottom ranked entries in the sorted list ‘L’ correspond to ‘dominant taxa’, when the list L is sorted in the increasing order of the abundance values. Similarly, one or more top ranked entries in the sorted list ‘L’ correspond to ‘dominant taxa’, and the one or more bottom ranked entries in the sorted list ‘L’ correspond to ‘sparse taxa’ when the list L is sorted in the decreasing order of the abundance values.

Sorted list (L) for the hypothetical microbiome sample (S1) indicated in Supplementary Table 1 is depicted below:

$$L\ (S1) = \{1, 1, 1, 1, 76, ..., 9852, 11974\}$$

In the set depicted above, the list L is sorted in the increasing order of the abundance values of individual microbes in the microbiome sample (S1). Consequently, one or more top ranked entries (i.e. beginning from the left hand side of the depicted lists) correspond to ‘sparse taxa’, and one or more bottom ranked entries (i.e. beginning from the right hand side of the depicted lists) correspond to ‘dominant taxa’.

Step 3 Count the minimum number of 'sparse taxa' (ST_i) whose cumulative abundance is ≥i% of the total population count 'M', when the abundance values are progressively cumulated in order of an **increasing order** of abundance value of each microbe in the sorted list 'L,' when the list L is sorted in **the increasing** order.

Step 3(a) Considering a value of i=1, the minimum number of 'Sparse taxa' (ST_1) which comprise ≥1% of the Total Population Count 'M' (wherein the sorted microbial abundances are cumulated in **increasing order** of abundance value of each microbe) in the hypothetical microbiome sample (S1) is depicted below.

S1 = {**1, 1, 1, 1, 76, 241, 745,** 1027, 2786, 3598, 4863, 6721, 7458, 9852, 11974}

In the above sample the cumulative abundance (1066) of the first 7 taxa (highlighted in boldface fonts) comprises ≥ 1% of Total Population Count (i.e. 49345).

Consequently, ST_1 for S1 = 7

Step 3(b) Similarly, considering a value of i=2, the minimum number of 'Sparse taxa' (ST_2) which comprise ≥2% of the Total Population Count 'M' (wherein the sorted microbial abundances are cumulated in **increasing order** of abundance value of each microbe) in the hypothetical microbiome sample (S1) is depicted below.

S1 = {**1, 1, 1, 1, 76, 241, 745,** 1027, 2786, 3598, 4863, 6721, 7458, 9852, 11974}

In the above sample the cumulative abundance (1066) of the first 7 taxa (highlighted in boldface fonts) comprises ≥ 2% of Total Population Count (i.e. 49345). Consequently, ST_2 for S1 = 7

Step 3(c) Similarly, considering a value of i=3, the minimum number of ‘Sparse taxa’ (ST_3) which comprise ≥3% of the Total Population Count 'M' (wherein the sorted microbial abundances are cumulated in **increasing order** of abundance value of each microbe) in the hypothetical microbiome sample (S1) is depicted below.

S1 = {**1, 1, 1, 1, 76, 241, 745, 1027**, 2786, 3598, 4863, 6721, 7458, 9852, 11974}

In the above sample the cumulative abundance (2093s) of the first 8 taxa (highlighted in boldface fonts) comprises ≥ 3% of Total Population Count (i.e. 49345).

Consequently, ST_3 for S1 = 8

Step 3(d) The steps 3a, 3b, and 3c are repeated to obtain counts of STi wherein i comprises 50 integer values ranging from 1 to 50 (both 1 and 50 included). This generates a set ($ST_{SampleName}$) comprised of 50 values.

$$ST_{SampleName} = \{ ST_1, ST_2, ST_3, \ldots.., ST_{50} \}$$

ST_{S1} = {7, 7, 8, 8, 9, 9, 9, 9, 9, 10, 10, 10, 10, 10, 10, 10, 10, 11, 11, 11, 11, 11, 11, 11, 11, 11, 11, 12, 12, 12, 12, 12, 12, 12, 12, 12, 12, 12, 12, 12, 13, 13, 13, 13, 13, 13, 13, 13, 13, 13}

Step 4 Counting minimum number of ‘dominant taxa’ (DT_j) whose cumulative abundance is ≥j% of the total population count 'M', when the abundance values are progressively cumulated in order of an **increasing order** of abundance value of each microbe in the sorted list ‘L’, when the list L is sorted in **the increasing order**.

Step 4(a) Considering a value of j=1, the minimum number of ‘Dominant taxa’ (DT_1) which comprise ≥1% of the Total Population Count 'M' (wherein the sorted microbial abundances are cumulated in

increasing order of abundance value of each microbe) in the hypothetical microbiome sample (S1) is depicted below.

S1 = {1, 1, 1, 1, 76, 241, 745, 1027, 2786, 3598, 4863, 6721, 7458, 9852, **11974**}

In the above sample the cumulative abundance (11974) of the last 1 taxon (highlighted in boldface fonts) comprises ≥ 1% of Total Population Count (i.e. 49345).

Consequently, DT_1 for S1 = 1

Step 4(b) Similarly, considering a value of j=2, the minimum number of ‘Dominant taxa’ (DT2) which comprise ≥2% of the Total Population Count 'M' (wherein the sorted microbial abundances are cumulated in increasing order of abundance value of each microbe) in the hypothetical microbiome sample (S1) is depicted below.

S1 = {1, 1, 1, 1, 76, 241, 745, 1027, 2786, 3598, 4863, 6721, 7458, 9852, **11974**}

In the above sample the cumulative abundance (11974) of the last 1 taxon (highlighted in boldface fonts) comprises ≥ 2% of Total Population Count (i.e. 49345).

Consequently, DT_2 for S1 = 1

Step 4(c) The steps 4a and 4b are repeated to obtain counts of DT_i wherein j comprises 50 integer values ranging from 1 to 50 (both 1 and 50 included). This generates a set ($DT_{SampleName}$) comprised of 50 values each.

$DT_{SampleName}$ = {DT_1, DT_2, DT_3, ..., DT_{50}}

DT_{S1} = {1, 1, 1, 1, 1, 1, 1, 1, 1, 1, 1, 1, 1, 1, 1, 1, 1, 1, 1, 1, 1, 1, 1, 1, 2, 2, 2, 2, 2, 2, 2, 2, 2, 2, 2, 2, 2, 2, 2, 2, 2, 2, 2, 2, 3, 3, 3, 3, 3, 3}

Step 5 Computing a Cartesian product of the sets 'DT' and 'ST' to obtain a set 'C' containing a plurality of ordered pairs corresponding to DTj and STi values.

For example, for sample S1, set $\mathbf{C}^{\mathbf{S1}}$is obtained in the following manner

DT^{S1} = {1, 1, 1, 1, 1, 1, 1, 1, 1, 1, 1, 1, 1, 1, 1, 1, 1, 1, 1, 1, 1, 1, 1, 1, 2, 2, 2, 2, 2, 2, 2, 2, 2, 2, 2, 2, 2, 2, 2, 2, 2, 2, 2, 2, 3, 3, 3, 3, 3, 3}

ST^{S1} = {7, 7, 8, 8, 9, 9, 9, 9, 9, 10, 10, 10, 10, 10, 10, 10, 10, 11, 11, 11, 11, 11, 11, 11, 11, 11, 11, 12, 12, 12, 12, 12, 12, 12, 12, 12, 12, 12, 12, 12, 13, 13, 13, 13, 13, 13, 13, 13, 13, 13}

$\mathbf{C}^{\mathbf{S1}}$ = Cartesian product of sets DT^{S1} and ST^{S1}

	DT_1	DT_2	DT_3	DT_4	DT_5	DT_6	DT_7	DT_8	.....	.....	DT_{50}
ST_1	(1,7)	(1,7)	(1,7)	(1,7)	(1,7)	(1,7)	(1,7)	(1,7)	.....	.....	(3,7)
ST_2	(1,7)	(1,7)	(1,7)	(1,7)	(1,7)	(1,7)	(1,7)	(1,7)	.....	.....	(3,7)
ST_3	(1,8)	(1,8)	(1,8)	(1,8)	(1,8)	(1,8)	(1,8)	(1,8)	.....	.....	(3,8)
ST_4	(1,8)	(1,8)	(1,8)	(1,8)	(1,8)	(1,8)	(1,8)	(1,8)	.....	.....	(3,8)
ST_5	(1,9)	(1,9)	(1,9)	(1,9)	(1,9)	(1,9)	(1,9)	(1,9)	.....	.....	(3,9)
ST_6	(1,9)	(1,9)	(1,9)	(1,9)	(1,9)	(1,9)	(1,9)	(1,9)	.....	.....	(3,9)
ST_7	(1,9)	(1,9)	(1,9)	(1,9)	(1,9)	(1,9)	(1,9)	(1,9)	.....	.....	(3,9)
ST_8	(1,9)	(1,9)	(1,9)	(1,9)	(1,9)	(1,9)	(1,9)	(1,9)	.....	.....	(3,9)
.....	.....	.....	.....	.....	.....	.....	.....	.....	.....	.....	.....
.....	.....	.....	.....	.....	.....	.....	.....	.....	.....	.....	.....
ST_{50}	(1,13)	(1,13)	(1,13)	(1,13)	(1,13)	(1,13)	(1,13)	(1,13)	.....	.....	(3,13)

Step 6 For each sample, a set ‘DSR’ is derived by computing (DTj ÷ STi) for each ordered pair in the set ‘C’

For example, for Sample S1, the set $\mathbf{DSR^{S1}}$ (represented below) is derived by computing (DTj ÷ STi) for each ordered pair in set $\mathbf{C^{S1}}$

	DT_1	DT_2	DT_3	DT_4	DT_5	DT_6	DT_7	DT_8	.....	.....	DT_{50}
ST_1	0.1429	0.1429	0.1429	0.1429	0.1429	0.1429	0.1429	0.1429	.....	.....	0.4286
ST_2	0.1429	0.1429	0.1429	0.1429	0.1429	0.1429	0.1429	0.1429	.....	.....	0.4286
ST_3	0.1250	0.1250	0.1250	0.1250	0.1250	0.1250	0.1250	0.1250	.....	.....	0.3750
ST_4	0.1250	0.1250	0.1250	0.1250	0.1250	0.1250	0.1250	0.1250	.....	.....	0.3750
ST_5	0.1111	0.1111	0.1111	0.1111	0.1111	0.1111	0.1111	0.1111	.....	.....	0.3333
ST_6	0.1111	0.1111	0.1111	0.1111	0.1111	0.1111	0.1111	0.1111	.....	.....	0.3333
ST_7	0.1111	0.1111	0.1111	0.1111	0.1111	0.1111	0.1111	0.1111	.....	.....	0.3333
ST_8	0.1111	0.1111	0.1111	0.1111	0.1111	0.1111	0.1111	0.1111	.....	.....	0.3333
.....	.....	.....	.....	.....	.....	.....	.....	.....	.....	.....	.....
.....	.....	.....	.....	.....	.....	.....	.....	.....	.....	.....	.....
ST_{50}	0.0769	0.0769	0.0769	0.0769	0.0769	0.0769	0.0769	0.0769	.....	.....	0.2308

Step 7 Computing a central tendency value (or a distribution characteristic) for the set 'DSR' obtained for each individual sample. In this example, median has been considered as the central tendency measure

Central tendency (Median) value of set $DSR^{S1} = 0.1538$

HOME

Step 8 Compute the 'Taxonomic Composition Skew' (TCS) by applying a mathematical transformation on the central tendency value. In this example, the mathematical transformation step comprises subtraction of the central tendency value from 1 (i.e. unity)

TCS **= 1 -** Central tendency value of **(DSR)**

TCS metric for S1 = 1 – median (DSR^{S1})

= 1 – 0.1538

= **0.8461**

References

1. Income inequality measures | Journal of Epidemiology & Community Health. Available at: http://jech.bmj.com/content/61/10/849.short. (Accessed: 22nd August 2017)
2. Kalmijn, W. Gini Coefficient. in *Encyclopedia of Quality of Life and Well-Being Research* (ed. Michalos, A. C.) 2559–2561 (Springer Netherlands, 2014). doi:10.1007/978-94-007-0753-5_1168
3. Gini coefficient - Oxford Reference. Available at: http://www.oxfordreference.com/view/10.1093/oi/authority.20110803095853429. (Accessed: 22nd August 2017)
4. Ellison, G. T. H. Letting the Gini out of the bottle? Challenges facing the relative income hypothesis. *Soc. Sci. Med. 1982***54,** 561–576 (2002).
5. Gold, R., Kawachi, I., Kennedy, B. P., Lynch, J. W. & Connell, F. A. Ecological analysis of teen birth rates: association with community income and income inequality. *Matern. Child Health J.***5,** 161–167 (2001).
